# Hospitalization risk of the 2009 H1N1 pandemic cases in Hong Kong

**DOI:** 10.1186/1471-2334-14-32

**Published:** 2014-01-16

**Authors:** Xi-Ling Wang, Chit-Ming Wong, Kwok-Hung Chan, King-Pan Chan, Pei-Hua Cao, JS Malik Peiris, Lin Yang

**Affiliations:** 1 School of Public Health, The University of Hong Kong, Hong Kong Special Administrative Region, People’s Republic of China; 2 Department of Microbiology, The University of Hong Kong, Hong Kong Special Administrative Region, People’s Republic of China; 3 HKU-Pasteur Center, Hong Kong Special Administrative Region, People’s Republic of China; 4 Squina International Centre for Infection Control, School of Nursing, The Hong Kong Polytechnic University, Hong Kong Special Administrative Region, People’s Republic of China; 5 5/F William Mong Block, 21 Sassoon Road, Pokfulam, Hong Kong Special Administrative Region

**Keywords:** Influenza, Pandemic, Hospitalization

## Abstract

**Background:**

Reliable assessment for the severity of the 2009 H1N1 pandemic influenza is critical for evaluation of vaccination strategies for future pandemics. This study aims to estimate the age-specific hospitalization risks of the 2009 pandemic cases during the first wave in Hong Kong, by combining the findings from the serology and disease burden studies.

**Methods:**

Excess hospitalization rates associated with the pandemic H1N1 were estimated from Poisson regression models fitted to weekly total numbers of non-accidental hospitalization from 2005 to 2010. Age-specific infection-hospitalization risks were calculated as excess hospitalization rates divided by the attack rates in the corresponding age group, which were estimated from serology studies previously conducted in Hong Kong.

**Results:**

Excess hospitalization rate associated with pandemic H1N1 was highest in the 0–4 age group (881.3 per 100,000 population), followed by the 5–14, 60+, 15–29, 50–59, 30–39 and 40–49 age groups. The hospitalization risk of the infected cases (i.e. infection-hospitalization risk) was found highest in the 60+ age group and lowest in the 15–29 age group, with the estimates of 17.5% and 0.7%, respectively.

**Conclusions:**

People aged 60 or over had a relatively high infection-hospitalization risk during the first wave of the 2009 H1N1 pandemic, despite of a low attack rate in this age group. The findings support the policy of listing older people as the priority group for pandemic vaccination.

## Background

The 2009 influenza pandemic caused by a novel swine-origin influenza A/H1N1 virus (A(H1N1)pdm09) was the first pandemic in the twenty-first century. Proper assessment for the severity of the 2009 H1N1 pandemic is critical for evaluation of vaccination strategies for future pandemics. Hospitalization risk of the infected cases, termed as "infection-hospitalization risk (IHR)" hereafter, is a key indicator for the severity of infectious diseases [1]. However obtaining a reliable estimate for IHR remains challenging, because infected cases might have not shown any symptoms or not been differentiated from other respiratory infections. Another challenge lies in a variety of numerators and denominators used for IHR calculation. Symptomatic infection-hospitalization risk (sIHR), which used the number of cases with influenza-like illness (ILI) as denominator and hospitalized cases with laboratory-confirmed A(H1N1)pdm09 as numerator, was quickly calculated at the early stage of the pandemic to allow timely medical resource allocation for the containment [2]. But sIHR could be a biased indicator, as not all the ILI cases were infected with A(H1N1)pdm09. Confirmed infection-hospitalization risk (cIHR) was later calculated as the number of hospitalized cases with laboratory-confirmed A(H1N1)pdm09 divided by the number of A(H1N1)pdm09 cases estimated from the serological studies on the seroconversion rates of A(H1N1)pdm09 antibodies among the general population [3-5]. The denominator of cIHR was able to capture asymptomatic and mild infections who did not seek medical treatment. However, the numerator of cIHR probably underestimated the true numbers of A(H1N1)pdm09 associated hospitalization, as not all the hospitalized cases were tested in a timely manner and some were tested negative due to waning virus titers when admitted into hospital several days after the onset of illness. To obtain reliable estimates of A(H1N1)pdm09-associated hospitalization, we conducted a study to calculate A(H1N1)pdm09-associated excess hospitalization, using a statistical model that has been widely applied in estimating disease burden of influenza. Model derived excess hospitalization is believed to capture both laboratory confirmed influenza hospitalized cases and those who were not laboratory diagnosed [6]. We then used this estimate of A(H1N1)pdm09-associated excess hospitalization as the numerator and the estimate of A(H1N1)pdm09 infected cases from the previous serological studies as the denominator [4,5], to calculate the infection-excess hospitalization risk (eIHR) of the 2009 H1N1 pandemic in Hong Kong.

## Methods

### Data

Hospitalization records from 2005 to 2010 in Hong Kong were obtained from the electronic database of the Hospital Authority, which manages 41 public hospitals covering 78% of total hospital bed-days in the whole territory [7]. These hospitalization data recorded up to 15 discharge diagnoses for each hospitalized patient, but were not linked to any baseline characteristics of lifestyle factors and co-morbidities nor specific medical treatments received by these patients. Weekly numbers of non-accidental hospitalization were aggregated for the age groups of 0–4, 5–14, 15–29, 30–39, 40–49, 50–59 and 60 or over (60+) years by excluding the cases with any of the 15 listed discharge diagnosis of accidental cause (International Classification of Diseases version 9 codes (ICD9), 001–799). Age-stratified laboratory-confirmed hospitalization with A(H1N1)pdm09 infection during the first wave pandemic (26 April 2009 to 2 January 2010) were obtained from the eFlu database managed by the Hospital Authority, which collected the demographic data as well as hospitalization and fatal outcomes of laboratory-confirmed A(H1N1)pdm09 cases in Hong Kong [5].

Influenza virus surveillance data were obtained from the microbiology laboratory of the Queen Mary Hospital, which is one of the largest public hospitals in Hong Kong. Nasopharyngeal specimens were collected from the patients with influenza-like symptoms and tested for influenza (type A and B), respiratory syncytial virus (RSV), adenovirus and parainfluenza viruses by immunofluorescence tests [8]. Nearly 90% of influenza A positive specimens were further subtyped into seasonal A(H1N1) and A(H3N2) by viral culture and haemagglutination inhibition tests. During the 2009 pandemic, the specimens were also tested for A(H1N1)pdm09 by RT-PCR. This laboratory tested all the virus samples collected in Hong Kong Island and provided more than 20% of specimens to the Department of Health virology surveillance network from 2005 to 2010. Our previous study has demonstrated that the virology data of this single laboratory were able to represent the virus activity in the entire Hong Kong [9]. The virology data were then aggregated into weekly age-specific numbers of positive specimens for each virus. Meteorological data of temperature and relative humidity were obtained from the Hong Kong Observatory.

### Statistical analysis

Poisson regression models were fitted to weekly numbers of non-accidental hospitalization for the age groups of 0–4, 5–14, 15–29, 30–39, 40–49, 50–59 and 60 or over (60+) years, with the influenza proxy variables of weekly age-specific numbers of positive specimens for A(H1N1), A(H3N2), B and A(H1N1)pdm09 simultaneously entered. Confounding was adjusted for by adding weekly age-specific positive isolate numbers of other respiratory viruses (RSV, adenovirus and parainfluenza), long term and seasonal trends, temperature and relative humidity, as previously described [10]. Baseline hospitalization was estimated by setting the proxy variable of A(H1N1)pdm09 to zero in the Poisson model under the assumption of no A(H1N1)pdm09 circulating. Age-specific excess hospitalization was then derived as the difference between the fitted and baseline hospitalization during the first pandemic wave of 26 April 2009 to 2 January 2010. Excess hospitalization rate was calculated by dividing excess hospitalization with age-specific annual population size, which was estimated from the census data by linear interpolation.

Age-specific eIHR was calculated with the age-specific excess hospitalization rate as numerator and the corresponding attack rate estimated from two local serological studies as denominator ( $\text{eIHR}=\frac{\text{Excess hospitalization rate}}{\text{Attack rate}}$) [4,5]. cIHR had a similar formula, in that the numerator was switched to laboratory confirmed A(H1N1)pdm09 hospitalization which was reported by the eFlu database of Hospital Authority ( $\text{cIHR}=\frac{\text{Laboratory confirmed hospitalization rate}}{\text{Attack rate}}$). Because the age-specific attack rate was only available for the first wave of the 2009 pandemic, we calculated the eIHR and cIHR only for this period. Also the attack rate of A(H1N1)pdm09 in young children aged below 5 years was not available in these serological studies; therefore we were unable to provide estimates for the 0–4 and all-ages groups. The 95% confidence interval of eIHR was calculated using the Delta method.

### Sensitivity analysis

Respiratory and circulatory diseases were considered as more specific outcomes to influenza infection than non-accidental hospitalization, although previous studies have shown that people with immunity-compromised chronic diseases (such as diabetes, renal diseases and cancers) also had higher risk of hospitalization and mortality associated with influenza [11,12]. Nevertheless, to test for the robustness of our models, we conducted a sensitivity analysis by replacing the outcome of non-accidental hospitalization with the more specific health outcome of respiratory and circulatory hospitalization. Similar to our main analysis, Poisson regression models were fitted to weekly numbers of respiratory and circulatory hospitalization for seven age groups, respectively.

The ethical approval for this study was obtained from the Institutional Review Board of the University of Hong Kong/Hospital Authority Hong Kong West Cluster (UV11-264). All analyses were conducted in the R package (version 2.14.2).

## Results

**Figure 1:**
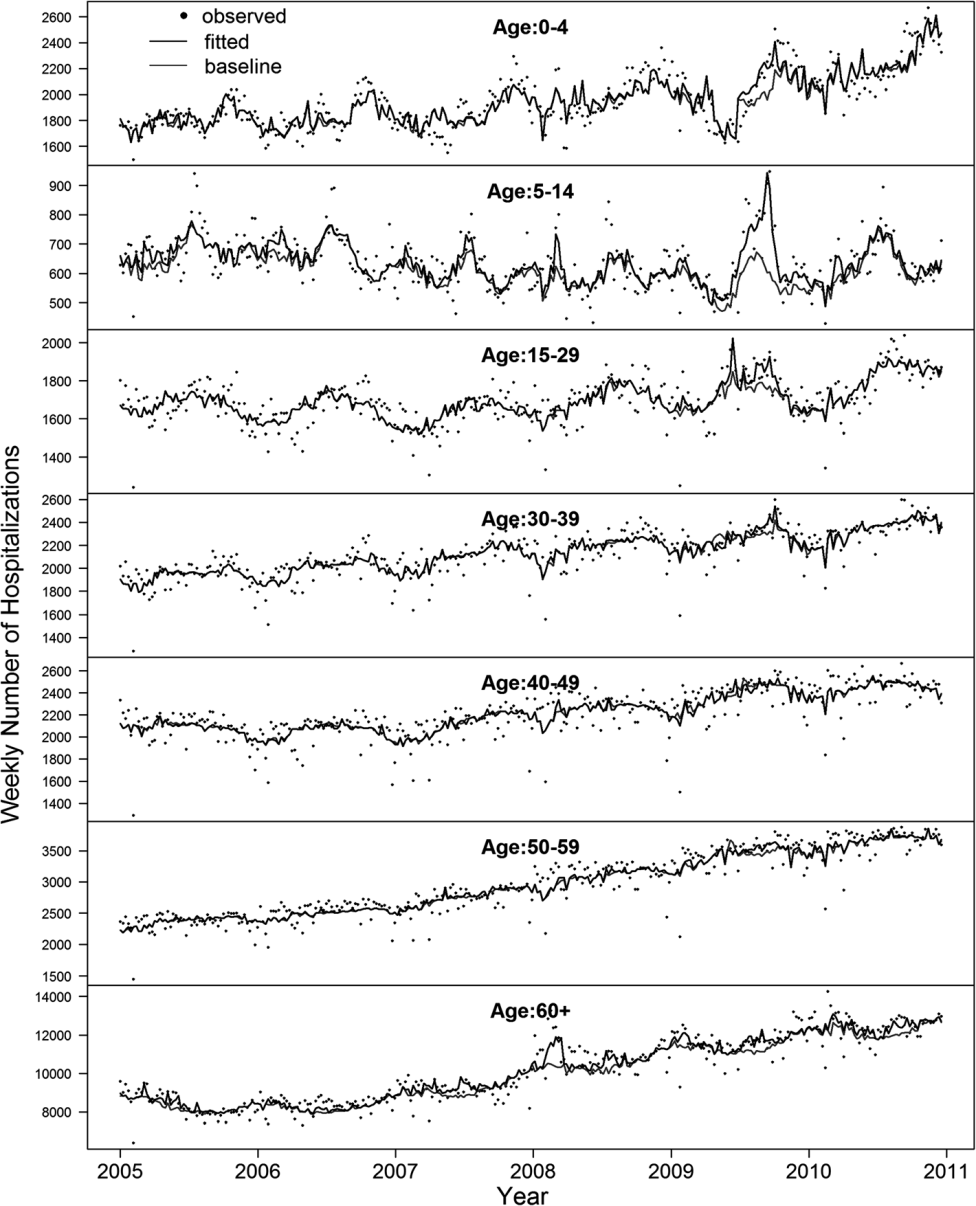
**Weekly observed (dots) and fitted (solid black line) numbers of hospitalization for non-accidental causes.** Solid grey line indicates the baseline when all the influenza proxies were assumed equal to zero.

Weekly observed and fitted non-accidental hospitalization number was shown in Figure 1. Significant association of A(H1N1)pdm09 with hospitalization was only found in the age groups below 30 years old (p < 0.05). According to our point estimate, during the first wave of the 2009 H1N1 pandemic, there were a total of 10,178 excess hospitalization associated with A(H1N1)pdm09, which was markedly higher than the total number of 7,386 laboratory-confirmed cases who were hospitalized and reported by the eFlu database. Excess rate of hospitalization associated with A(H1N1)pdm09 was highest in the 0–4 age group (881.3 per 100,000 population), followed by the 5–14, 60+, 15–29, 50–59, 30–39 and 40–49 age groups (Table 1). The estimated excess rates were comparable to the rates of laboratory confirmed hospitalization in children and young adults, but one to three times higher for the age groups of 50–59 and 60+. The eIHR were found highest in the 60+ age group and lowest in the 15–29 age group, with the estimates of 17.5% (95% confidence interval -64.4%, 99.4%) and 0.7% (0.2%, 1.2%), respectively. eIHR ranged from 0.9% to 1.6% in the rest of age groups (Table 1).

**Table 1:** Infection-hospitalization risks based on confirmed hospitalization (cIHR) and on excess hospitalization (eIHR) during the first wave of the 2009 H1N1 pandemic

**Age**	**Population**	Infection attack rate^*^		**Confirmed Hospitalization**		**cIHR**		**Excess Hospitalization**		**eIHR**	
		**%**	**(95% CI)**	**No.**	**Rate(per 100,000 population)**	**%**	**(95% CI)**	**No.**	**Rate(per 100,000 population)**	**%**	**(95% CI)**
**0-4**	234583	NA		1842	785.2	NA		2067	881.3	NA	
**5-14**	635423	43.5	(39.6, 48.3)	2445	384.8	0.9	(0.8, 1.0)	2774	436.6	1.0	(0.0, 2.0)
**15-29**	1410126	16.9	(12.4, 21.3)	1313	93.1	0.6	(0.4, 0.8)	1650	117.0	0.7	(0.2, 1.2)
**30-39**	1118446	5.8	(3.1, 9.7)	470	42.0	0.7	(0.4, 1.4)	554	49.6	0.9	(-2.2, 3.9)
**40-49**	1267108	3.8	(1.1, 7.5)	375	29.6	0.8	(0.4, 2.7)	501	39.5	1.0	(-3.6, 5.7)
**50-59**	1073032	5.0	(2.4, 8.3)	495	46.1	0.9	(0.6, 1.9)	878	81.8	1.6	(-3.3, 6.5)
**60+**	1249965	0.8	(0.2, 4.2)	446	35.7	4.5	(0.8, 17.8)	1754	140.3	17.5	(-64.4, 99.4)

Abbreviations: *CI* confidence interval, *NA* not available.

* Adapted from ref (*4,5*).

The sensitivity analysis showed that A(H1N1)pdm09-associated hospitalization for respiratory and circulatory diseases accounted for 65-89% of those for non-accidental hospitalization in all the age groups, with an age pattern similar to the non-accidental hospitalization (data not shown).

## Discussion

Our study applied the Poisson regression model rather than the Serfling approach to estimate the influenza-associated hospitalization in Hong Kong. The Serfling approach requires a clear seasonality of influenza virus activity to define non-epidemic and epidemic period [13]. However, this prerequisite cannot be satisfied in subtropical and tropical regions like Hong Kong, where seasonality of influenza is less clear and influenza virus circulates throughout the year [14]. Moreover, it is difficult to separate the effects of multiple respiratory viruses using the Serfling approach as the seasonal peaks of many viruses tended to overlap with each other. On the contrary, the Poisson regression model does not require a clear seasonal pattern of influenza, thus it is especially useful for tropical and subtropical regions [14]. In the Poisson regression model, we used the age-specific positive number as a proxy for the activity of each virus. During the pandemic, the laboratory practice could be biased towards children and young adults as they were more likely to be tested, so the all-ages positive number might be a biased indicator for virus activity. The use of age-specific positive number ensured that the changes in laboratory practice between age groups would not affect our age-specific results. Our models were partly validated by comparing the model estimates with the laboratory-confirmed cases of A(H1N1)pdm09 infections, which was reported by the electronic reporting system eFlu managed by the Hospitalization Authority. These reported numbers could be regarded as the lower boundary of the hospitalization burden caused by the pandemic. Our model estimates were all above these lower bounds, suggesting that our estimates were valid.

The cumulative incidence of A(H1N1)pdm09 from serologic study revealed high attack rates in children and adolescents, with almost half (43.5%) of school age children infected during the first wave of the 2009 H1N1 pandemic. However the attack rate in the older population was much lower, with only 0.8% of people aged 60 or over infected. This pattern of attack rates was consistent with findings from a British serologic survey [3]. The different attack rates across age groups could be probably due to their prior exposure to antigenically similar influenza viruses [15]. Here we used serological attack rates as the denominator of IHR, which could provide a better assessment for morbidity risk after infection than one using influenza-like-illness incidence as the denominator, as the former could include asymptomatic infections and minimize the ascertainment bias.

Early sIHR estimates in the US showed a higher hospitalization risk in the 0–4 age group than the other age groups [2]. The estimates of all-ages sIHR or cIHR later reported a range from 0.14% to 1% [16,17]. Our findings of higher eIHR in the 50–59 and 60+ age groups echoed a study in Netherlands, although their estimates of cIHR were markedly lower than ours [18]. Higher hospitalization risks in Hong Kong could probably be explained by regional difference in health seeking behavior and host immunity levels. Based on our previous estimate of 127 deaths associated with the pandemic influenza during the same period [9], the hospitalization-fatality risk for the first wave of the 2009 pandemic was around 1% in Hong Kong, suggesting a relatively mild pandemic. At the beginning of the 2009 pandemic, the Hong Kong Special Administrative Region government issued instant warnings and at the same time quickly adopted a variety of nonpharmaceutical control measures, such as school closure and border control, in the whole territory [19]. Special influenza clinics were soon open and laboratory tests were extensively conducted, especially in children and young adults who were immediately identified as high risk groups. The close match of eIHR and cIHR point estimates, together with the low mortality rate in all the age groups younger than 50 years [9,12], suggests that these control measures might have effectively identified the infected cases and successfully reduced casualties among young people.

Our eIHR estimates revealed a higher hospitalization risk of A(H1N1)pdm09 cases in people aged over 60 years than the other age groups. Previous studies have demonstrated that during the first wave of the 2009 H1N1 pandemic, the attack rate of A(H1N1)pdm09 was lower in adults aged over 60 years, probably due to their preexisting antibodies against A(H1N1)pdm09 [20]. However, this does not mean older people were fully exempted from the severe complications caused by A(H1N1)pdm09 infections. Serious underreporting of A(H1N1)pdm09 cases in Hong Kong elders under medical settings, as revealed by our study [10] and also others, requires further investigations. This underreporting might be partly due to less typical influenza-like symptoms after the A(H1N1)pdm09 infections compared to younger adults [21], or chronic conditions commonly occurred in older population. Studies have found that the risk of hospitalization and ICU admission after A(H1N1)pdm09 infections markedly increased among people with chronic conditions such as cardiovascular, respiratory and metabolic conditions [22]. Our previous estimates on cause-specific hospitalization associated with A(H1N1)pdm09 also indicated that a large proportion of hospitalization occurred in those with chronic conditions [10]. It is of note that our point estimate of eIHR in the 60+ age group requires cautious interpretation because of its wide confidence intervals, probably due to the relative short period of pandemic in the first wave. Nevertheless, it is surprising that the eFlu surveillance system was able to capture the majority of hospitalized pandemic cases younger than 50, but only captured 30-60% hospitalized pandemic cases for those older than 50 years. The hidden burden in older population of Hong Kong reveals a need to enhance laboratory surveillance in this population in future pandemic.

Whether vaccination priority shall be given to people with high mortality/hospitalization risk or to those more likely transmit the virus during the pandemic remains a controversial issue. However, most health authorities adopted the former vaccination strategy with the aim to reduce casualty and severe outcomes. In Hong Kong, the pandemic vaccines were not available until the end of December 2009 and older people were immediately listed as the priority group for pandemic vaccination despite of the low attack rate in this age group during the pandemic. Our findings of higher eIHR in older population well support the decision by the health authority of Hong Kong and most of other countries [23,24]. However, if vaccines could be ready at the early phase of future pandemics, the decision on vaccination strategy should rely on quick and accurate estimates on transmission, morbidity and mortality burden in different age groups, in order to make the optimal utilization of medical resources.

## Conclusions

Our study found that during the first wave of the 2009 H1N1 pandemic, hospitalization risk of infected pandemic cases was higher in old people than in young adults and children. Old persons may require more medical resources after infection of the pandemic influenza. The findings support to include older people in the priority groups for pandemic vaccination.

## Competing interests

The authors declare that they have no competing interests.

## Authors’ contributions

Conceived and designed the experiments: LY CMW. Analyzed the data: XLW LY KPC PHC. Wrote the paper: XLW LY KPC PHC CMW JSMP KHC. Interpreted the results: LY CMW. All authors read and approved the final manuscript.

## Pre-publication history

The pre-publication history for this paper can be accessed here:

http://www.biomedcentral.com/1471-2334/14/32/prepub

## References

[B1] Kelly H Cowling BJ Case fatality: rate, ratio, or risk? Epidemiology 2013 14 4 622 623 10.1097/EDE.0b013e318296c2b6 23732740

[B2] Presanis AM De Angelis D Hagy A Reed C Riley S Cooper BS Finelli L Biedrzycki P Lipsitch M The severity of pandemic H1N1 influenza in the United States, from April to July 2009: a Bayesian analysis PLoS Med 2009 14 12 e1000207 10.1371/journal.pmed.1000207 19997612 PMC2784967

[B3] Miller E Hoschler K Hardelid P Stanford E Andrews N Zambon M Incidence of 2009 pandemic influenza A H1N1 infection in England: a cross-sectional serological study Lancet 2010 14 9720 1100 1108 10.1016/S0140-6736(09)62126-7 20096450

[B4] Riley S Kwok KO Wu KM Ning DY Cowling BJ Wu JT Ho LM Tsang T Lo SV Chu DK Ma ES Peiris JS Epidemiological characteristics of 2009 (H1N1) pandemic influenza based on paired sera from a longitudinal community cohort study PLoS Med 2011 14 6 e1000442 10.1371/journal.pmed.1000442 21713000 PMC3119689

[B5] Wu JT Ma ES Lee CK Chu DK Ho PL Shen AL Ho A Hung IF Riley S Ho LM Lin CK Tsang T Lo SV Lau YL Leung GM Cowling BJ Malik Peiris JS The infection attack rate and severity of 2009 pandemic H1N1 influenza in Hong Kong Clin Infect Dis 2010 14 10 1184 1191 10.1086/656740 20964521 PMC3034199

[B6] Thompson WW Shay DK Weintraub E Brammer L Cox N Anderson LJ Fukuda K Mortality associated with influenza and respiratory syncytial virus in the United States JAMA 2003 14 2 179 186 10.1001/jama.289.2.179 12517228

[B7] Hospital Authority Hospital Authority Statistical Report 2009–2010 2011 Hong Kong

[B8] Chan KH Maldeis N Pope W Yup A Ozinskas A Gill J Seto WH Shortridge KF Peiris JS Evaluation of the Directigen FluA + B test for rapid diagnosis of influenza virus type A and B infections J Clin Microbiol 2002 14 5 1675 1680 10.1128/JCM.40.5.1675-1680.2002 11980941 PMC130655

[B9] Yang L Chan KP Cowling BJ Chiu SS Chan KH Peiris JS Wong CM Excess mortality associated with the 2009 pandemic of influenza A(H1N1) in Hong Kong Epidemiol Infect 2012 14 9 1542 1550 10.1017/S0950268811002238 22074735

[B10] Yang L Wang XL Chan KP Cao PH Lau HY Peiris JS Wong CM Hospitalisation associated with the 2009 H1N1 pandemic and seasonal influenza in Hong Kong, 2005 to 2010 Euro Surveill: bulletin europeen sur les maladies transmissibles = European communicable disease bulletin 2012 14 45 20309 23153475

[B11] Wong CM Yang L Chan KP Leung GM Chan KH Guan Y Lam TH Hedley AJ Peiris JS Influenza-associated hospitalization in a subtropical city PLoS Med 2006 14 4 e121 10.1371/journal.pmed.0030121 16515368 PMC1391978

[B12] Wu P Goldstein E Ho LM Yang L Nishiura H Wu JT Ip DK Chuang SK Tsang T Cowling BJ Excess mortality associated with influenza A and B virus in Hong Kong, 1998–2009 J Infect Dis 2012 14 12 1862 1871 10.1093/infdis/jis628 23045622 PMC3502382

[B13] Serfling RE Methods for current statistical analysis of excess pneumonia-influenza deaths Public Health Rep 1963 14 6 494 506 10.2307/4591848 19316455 PMC1915276

[B14] Viboud C Alonso WJ Simonsen L Influenza in tropical regions PLoS Med 2006 14 4 e89 10.1371/journal.pmed.0030089 16509764 PMC1391975

[B15] Bautista E Chotpitayasunondh T Gao Z Harper SA Shaw M Uyeki TM Zaki SR Hayden FG Hui DS Kettner JD Kumar A Lim M Shindo N Penn C Nicholson KG Clinical aspects of pandemic 2009 influenza A (H1N1) virus infection N Engl J Med 2010 14 18 1708 1719 20445182 10.1056/NEJMra1000449

[B16] Bandaranayake D Huang QS Bissielo A Wood T Mackereth G Baker MG Beasley R Reid S Roberts S Hope V Risk factors and immunity in a nationally representative population following the 2009 influenza A(H1N1) pandemic PLoS One 2010 14 10 e13211 10.1371/journal.pone.0013211 20976224 PMC2954793

[B17] Dawood FS Hope KG Durrheim DN Givney R Fry AM Dalton CB Estimating the disease burden of pandemic (H1N1) 2009 virus infection in Hunter New England, Northern New South Wales, Australia, 2009 PLoS One 2010 14 3 e9880 10.1371/journal.pone.0009880 20360868 PMC2848017

[B18] Steens A Waaijenborg S Teunis PF Reimerink JH Meijer A van der Lubben M Koopmans M van der Sande MA Wallinga J van Boven M Age-dependent patterns of infection and severity explaining the low impact of 2009 influenza A (H1N1): evidence from serial serologic surveys in the Netherlands Am J Epidemiol 2011 14 11 1307 1315 10.1093/aje/kwr245 22025354

[B19] Cowling BJ Lau MS Ho LM Chuang SK Tsang T Liu SH Leung PY Lo SV Lau EH The effective reproduction number of pandemic influenza: prospective estimation Epidemiology 2010 14 6 842 846 10.1097/EDE.0b013e3181f20977 20805752 PMC3084966

[B20] Hancock K Veguilla V Lu X Zhong W Butler EN Sun H Liu F Dong L DeVos JR Gargiullo PM Brammer TL Cox NJ Tumpey TM Katz JM Cross-reactive antibody responses to the 2009 pandemic H1N1 influenza virus N Engl J Med 2009 14 20 1945 1952 10.1056/NEJMoa0906453 19745214

[B21] Pano-Pardo JR Viasus D Pachon J Campins A Lopez-Medrano F Villoslada A Gutierrez-Cuadra M Pumarola T del Toro MD Oteo JA Martinez-Montauti J Gutierrez-Aroca J Segura F Carratala J Pandemic 2009 A(H1N1) infection requiring hospitalization of elderly Spanish adults J Am Geriatr Soc 2012 14 4 740 744 10.1111/j.1532-5415.2012.03903.x 22462803

[B22] Van Kerkhove MD Vandemaele KA Shinde V Jaramillo-Gutierrez G Koukounari A Donnelly CA Carlino LO Owen R Paterson B Pelletier L Vachon J Gonzalez C Hongjie Y Zijian F Chuang SK Au A Buda S Krause G Haas W Bonmarin I Taniguichi K Nakajima K Shobayashi T Takayama Y Sunagawa T Heraud JM Orelle A Palacios E van der Sande MA Wielders CC Risk factors for severe outcomes following 2009 influenza A (H1N1) infection: a global pooled analysis PLoS Med 2011 14 7 e1001053 10.1371/journal.pmed.1001053 21750667 PMC3130021

[B23] Johansen K Nicoll A Ciancio BC Kramarz P Pandemic influenza A(H1N1) 2009 vaccines in the European Union Euro Surveill: bulletin europeen sur les maladies transmissibles = European communicable disease bulletin 2009 14 41 19361 19883538

[B24] Lau JT Yeung NC Choi KC Cheng MY Tsui HY Griffiths S Acceptability of A/H1N1 vaccination during pandemic phase of influenza A/H1N1 in Hong Kong: population based cross sectional survey BMJ 2009 14 b4164 10.1136/bmj.b4164 19861377 PMC2768779

